# Lempel-Ziv Complexity and Structural Features of DNA Methylation Reveal Epigenetic Rejuvenation in Mouse Embryogenesis

**DOI:** 10.3390/genes17080925

**Published:** 2026-08-06

**Authors:** Andrey Vl. Timofeev, Alexander Bratchikov, Alexander Anufriev

**Affiliations:** LLC “AI Center for SCO+ Countries”, St. Petersburg 197341, Russia; bav@centeraisco.com (A.B.); aas@centeraisco.com (A.A.)

**Keywords:** epigenetic rejuvenation, DNA methylation entropy, scNMT-seq, ground zero, information theory, persistent entropy, topological analysis

## Abstract

**Background**: DNA methylation is a key epigenetic mechanism whose dynamics are closely linked to ageing. Modern epigenetic clocks predict biological age based on the average methylation level. The concept of “epigenetic rejuvenation” posits that at early stages of development, the biological age of the embryo may decrease, reaching a minimum (“ground zero”) at the gastrulation stage. However, standard averaging methods may not account for important rearrangements in the internal structure of methylation. **Objective**: To apply the apparatus of information theory and topological data science to the analysis of scNMT-seq data and to test whether DNA methylation entropy decreases from stage E4.5 to E6.5, which would correspond to an approach towards the biological zero state. **Methods**: Publicly available scNMT-seq data (GSE121690) were analyzed. Five entropy measures were calculated for each cell (Shannon, Renyi, Tsallis, LZ-complexity, local gradient entropy (entropy of variations in the smoothed histogram of the methylation distribution), and persistent entropy (PE)—a topological complexity measure). For the five-dimensional entropy feature space, a Rips complex was constructed, and persistence diagrams H_0 and H_1 were computed. **Results**: All five entropy measures decreased significantly, with LZ complexity showing the largest relative reduction (−28.4%) and the strongest independent signal (partial r = −0.181). Among all the complexity measures studied, LZ complexity exhibited the largest relative reduction, underscoring its heightened sensitivity to the progressive ordering of the epigenetic landscape. Notably, the ternary encoding of LZ complexity showed strong correlation with Shannon entropy (r = 0.71), indicating that algorithmic complexity, when accounting for partial methylation states, aligns closely with statistical entropy while retaining sensitivity to spatial order. The consistency of results across binary and ternary encodings confirms the robustness of LZ complexity as a structural biomarker. Persistent entropy confirmed the general dynamics (decrease from 15.91 to 14.89, *p* = 0.01). Topological analysis of the multidimensional space revealed a qualitative reorganization: at stage E6.5, stable cyclic structures (H_1_) emerge, while at E4.5 the space is dominated by a single large-scale cycle. Null model validation confirmed that the observed H_1_-cycles are genuine topological features rather than random fluctuations. Comprehensive topological characterization showed that normalized persistent entropy increases from 0.846 to 0.882 (*p* < 0.001), while maximum persistence decreases from 0.446 to 0.218 (*p* < 0.001), reflecting a transition from a homogeneous state to structured diversification—multiple, evenly distributed cycles corresponding to distinct cell lineages. Consistent with this, regional disorder (RE/RD) at the single-cell level decreases from E4.5 to E6.5 (RE: −25.5%, RD: −27.4%, *p* < 10^−13^), while global entropy also decreases, together painting a picture of epigenetic rejuvenation as ordered consolidation at the whole-genome scale. An SVM model trained on 15 entropy and structural features achieved stage classification with an accuracy of 93.4% and AUC of 0.981, confirming the diagnostic potential of the approach. **Conclusions**: The decrease in DNA methylation entropy from E4.5 to E6.5 corresponds to an approach to “ground zero”—the point of minimum biological age in embryogenesis—and supports the hypothesis of a link between decreasing entropy and epigenetic rejuvenation. The addition of topological analysis reveals the hidden organization of epigenetic diversity, showing that ordering does not lead to homogenization but is accompanied by the formation of distinguishable cell lineages.

## 1. Introduction

The fundamental question of the biology of ageing is why new organisms do not inherit the age of their parent germ cells. Somatic cells age by accumulating damage, whereas the germline is “reset” in each generation [1,2]. Kerepesi and co-authors experimentally confirmed this by using epigenetic clocks to show that the biological age of the embryo decreases during early development, reaching a minimum at the gastrulation stage [3,4]. This point was termed “ground zero”—the beginning of organismal life and ageing [5,6].

Epigenetic clocks based on the average methylation level have become the gold standard for assessing biological age. They predict the development of age-associated diseases, including Alzheimer’s disease and cardiovascular pathologies [4,7]. However, the mean value is not a sufficient statistic for describing the methylation distribution, since it does not account for correlations between CpG sites and cannot distinguish functionally different epigenetic states characterized by the same methylation level. For example, at the same average methylation (0.5), the patterns “11110000” and “10100101” are indistinguishable by the mean value. Shannon entropy, based on state frequencies, also does not distinguish these patterns (both yield an entropy of 1.0 bit). However, LZ complexity is sensitive to the order of elements: for “11110000” it is considerably lower than for “10100101”, reflecting the presence of ordered blocks. This illustrates the necessity of combining statistical and algorithmic measures for a complete description of the epigenetic landscape. Recent studies have shown that methylation entropy may serve as an additional biomarker of ageing: Chan et al. (2025) demonstrated that on the basis of entropy at 3000 CpG loci in buccal cells, chronological age can be predicted with an error of ~5 years, with entropy changes proving to be locus-specific and bidirectional—entropy of a number of regions decreased with age, whereas that of others increased [8]. Experiments on editing individual CpG sites (Liesenfelder et al., 2025) showed that modification of a single locus can affect the methylation of hundreds of linked regions, indicating the non-local character of the epigenetic network [7]. Singh (2025) proposed a theoretical justification for the link between methylation entropy and chromatin phase separation via the Flory–Huggins parameter [9]. Such non-local influence of one CpG site on hundreds of others points to the network organization of the epigenetic landscape and confirms that DNA methylation is not merely a set of independent marks but a complex information system with long-range correlations.

We propose to consider the epigenetic code as a language. The methylation level is the presence/absence of a “letter”, and entropy is a measure of the randomness of the text. High entropy is meaningless noise; low entropy is a structured message (“blank slate”).

The aim of this work is to test whether structural and algorithmic complexity measures—particularly LZ complexity—capture epigenetic ordering during natural rejuvenation, beyond what is reflected by mean methylation or Shannon entropy alone. Unlike Shannon entropy, which is partially confounded by mean methylation in low-coverage data, LZ complexity measures the spatial arrangement of methylation patterns and provides independent information about epigenetic organization.

## 2. Materials and Methods

### 2.1. Data

Publicly available scNMT-seq (single-cell methylation and accessibility) data from the NCBI GEO database (GSE121690), Bethesda, MD, USA [10] were used. The data contain 364 mouse cells at developmental stages E4.5 (267 cells) and E6.5 (97 cells). Each file contains information on CpG-site methylation levels in the format: chr, pos, met_reads, nonmet_reads, rate (methylation level from 0 to 1). scNMT-seq is a method enabling simultaneous profiling of DNA methylation, chromatin accessibility, and the transcriptome in a single cell [11].

### 2.2. Entropy Measures

For each cell, the vector of CpG-site methylation levels (rate values from 0 to 1) was discretized using a histogram with a fixed number of bins (B) over the range [0, 1]. For the resulting probability distribution $P=\{p_{1},p_{2},\ldots ,p_{B}\}$, where $p_{i}$ is the fraction of CpG sites falling into the i-th bin, the following measures were calculated. The choice of B = 20 bins is based on a trade-off between distribution resolution and statistical stability of estimates: with an average of approximately 400,000 CpG sites per cell, each bin contains roughly 20,000 sites, ensuring reliable probability estimation and being consistent with previous work on methylation entropy [8].

**Shannon entropy**—a classical diversity measure characterizing the uncertainty of a distribution:

$$H_{S}=-\sum_{i=1}^{B}p_{i}{log}_{2}p_{i}$$

**Renyi entropy** (of order α)—a generalization of Shannon entropy that, at α = 2, gives greater weight to frequently occurring patterns, suppressing the contribution of rare events (collision entropy):

$$H_{2}=-{log}_{2}\left(\sum_{i=1}^{B}p_{i}^{2}\right)$$

**Tsallis entropy** (with parameter q = 2)—a non-additive measure used for analyzing complex systems with long-range interactions:

$$S_{2}=\frac{1-\sum_{i=1}^{B}p_{i}^{2}}{q-1}$$

**LZ complexity (Lempel-Ziv complexity) [12]**—a measure of algorithmic complexity that estimates the number of unique substrings in a sequence. Unlike statistical measures (Shannon, Renyi, and Tsallis entropy), which operate on probability distributions, LZ complexity characterizes structural diversity and the degree of pattern repetitiveness in a specific realization of a sequence.

The LZ complexity calculation algorithm is as follows.

For the calculation, the original vector of methylation levels $r=\left(r_{1},r_{2},\ldots ,r_{N}\right)$, where $r_{j}\in \left[0,1\right]$, was binarised with a threshold of 0.5:

$$x_{j}=\left\{\begin{array}{ll} 1, & r_{j}>0.5\\ 0, & r_{j}\leq 0.5 \end{array}\right.$$

Note that CpG-site methylation levels represent a continuous stochastic process with discrete measurements (typically 5 levels: 0, 0.25, 0.5, 0.75, 1.0). However, for the present study, aimed at identifying qualitative patterns (entropy reduction, stage-specific correlations with repair genes), the use of a deterministic approximation (binarization with a threshold of 0.5) is a justified and standard approach in the analysis of scNMT-seq data. Binarisation serves as a tool for transitioning to a discrete representation for the calculation of algorithmic complexity (LZ) and topological measures. As a result, a binary string $x=(x_{1},x_{2},\ldots ,x_{N})$, where $x_{j}\in \{0,1\}$, is obtained. LZ complexity is defined in the process of lexicographic parsing of the string according to the following rule:

Let $x=(x_{1},x_{2},\ldots ,x_{N})$ be the string under analysis. The LZ complexity $c\left(x\right)$ equals the number of steps required to construct the entire string by sequentially adding new substrings that have not previously occurred in the already constructed part.

LZ parsing algorithm (classical Lempel-Ziv algorithm):

1. Initialisation: c=1, current string $Q=x_{1}$ (first character). In this phase, we begin with element $s_{1}$.
2. For each subsequent symbol $x_{i}$ (starting from i=2):
  - A trial string $Q^{\prime }=Q+x_{i}$ is formed (concatenation of the current string and the new symbol).
  - If $Q^{\prime }$ does not occur as a substring in the already constructed part of the string (i.e., in the concatenation of all previously extracted unique blocks), then:
    - c=c+1 (a new unique block is registered);
    - $Q=x_{i}$ (a new current string is started from this symbol).
  - Otherwise (if $Q^{\prime }$ has already occurred):
    - $Q=Q^{\prime }$ (the current string is extended without incrementing the counter).
3. If after processing all symbols Q is not empty, then c=c+1.

By the “already constructed part of the string” we mean the concatenation of all unique substrings (blocks) that were extracted at previous steps of the algorithm. It is against this accumulated string that the trial extension $Q^{\prime }$ is compared. If $Q^{\prime }$ has already occurred as a contiguous substring in the accumulated string, then it is not new, and we simply extend Q. If it has not occurred, a new block is fixed and the next one is begun. This is the standard implementation of LZ parsing.

**Ternary LZ complexity.** To account for hemimethylated states (rate ≈ 0.5), which may reflect epigenetic plasticity, we additionally computed LZ complexity using a ternary alphabet. Methylation rates were discretized as follows:

$$x_{j}=\left\{\begin{array}{cc} 0, & r_{j}<0.4\\ 1, & 0.4\leq r_{j}\leq 0.6\\ 2, & r_{j}>0.6 \end{array}\right.$$

The value 1 represents the hemimethylated/uncertain state. Normalization was performed as:

$$LZ_{norm}=\frac{LZ}{N/{log}_{k}(N)}$$

where k=3 for a ternary alphabet. This normalization ensures comparability across cells with different numbers of CpG sites. The use of ternary encoding preserves information about partial methylation, which is lost in binary binarization, and provides a more biologically realistic estimate of algorithmic complexity.

**Interpretation.** LZ complexity reflects the presence of repeating structures in a sequence. High values correspond to complex, poorly compressible sequences with a large number of unique patterns. Low values correspond to sequences with a high degree of repetitiveness (well compressible). In the context of DNA methylation, this means that LZ complexity estimates how chaotically methylated and unmethylated sites alternate along the genome. Ordered patterns (e.g., long blocks of “000…000111…111”) are characterized by low LZ complexity, whereas chaotic intermixing (“010101…”)—by high LZ complexity.

**Distinction from other entropy measures.** If Shannon, Renyi, and Tsallis entropy assess the probability distribution of states (ignoring the order of occurrence), then LZ complexity accounts for sequence and is sensitive to the spatial organization of patterns.

**Spatial entropy (entropy of local gradients)**. This is a measure that accounts for the order of methylation levels along the genome. In other words: the entropy of variations in the smoothed histogram of the methylation distribution. It was calculated from the variation in the smoothed histogram of the methylation distribution:

$$S_{spatial}=-\sum_{j=1}^{B-1}\Delta_{j}{log}_{2}(\Delta_{j}+\varepsilon )$$

where $\Delta_{j}=|{\overset{ˉ}{p}}_{j+1}-{\overset{ˉ}{p}}_{j}|$, and $p_{i}$ is the histogram value smoothed by a moving average with window 3, $\varepsilon =10^{-10}$ is a small correction avoiding the logarithm of zero. This measure is sensitive to local changes in methylation density, characterizing the degree of “intermixing” of methylated and unmethylated regions.

**Persistent entropy (PE)**. This is a topological complexity measure based on analysis of the persistence diagram H_0 constructed from the binarized methylation sequence (threshold 0.5). For the calculation, one-dimensional cubical filtration implemented in the GUDHI library [13] is used. Let there be m finite intervals (connected components) with lifetimes (persistences) $l_{1},l_{2},\ldots ,l_{m}$. The total sum of persistences is $L=\sum_{i=1}^{m}l_{i}$, and the normalized weights are $p_{i}=l_{i}/L$. Then persistent entropy is computed by the Shannon formula:

$$PE=-\sum_{i=1}^{m}p_{i}{log}_{2}p_{i}$$

PE characterizes the diversity of scales of structural elements in the methylation distribution: low values correspond to the dominance of extended homogeneous regions, high values—the presence of blocks of varying sizes. Unlike statistical entropies, PE accounts for spatial order and multiscale block structure, and unlike LZ complexity, it distinguishes the contribution of short and long blocks. For example: for the sequence “000111000111”, two blocks of methylated sites have lengths (persistences) 3 and 3 (the blocks are completely isolated). If the blocks were of different lengths, say 3 and 6, PE would reflect this difference. For equal blocks (3,3), PE = 1.0 bit (maximum for two blocks). With the dominance of one long block (e.g., 1 and 8), PE would be lower (≈0.42). Thus, PE distinguishes situations where short and long blocks make different contributions to structural complexity.

### 2.3. Statistical Analysis

Comparison of entropy between stages was performed using a parametric *t*-test and a non-parametric Mann–Whitney U test. The joint use of both tests allows the significance of differences to be confirmed regardless of whether the assumption of normality of distributions holds. Correlation analysis was performed using Pearson’s correlation coefficient. To control for multiple comparisons in the analysis of six entropy measures, false discovery rate (FDR) correction by the Benjamini–Hochberg method was applied. *p*-values were ordered in ascending order: $p_{\left(1\right)}\leq p_{\left(2\right)}\leq \cdots \leq p_{\left(m\right)}$, where m=6 is the number of tested hypotheses. Adjusted q-values were computed by the formula:

$$q_{\left(i\right)}=\underset{j\geq i}{min}\;\left(min(p_{\left(j\right)}\frac{m}{j},\;1)\right),$$

with monotonic non-increasing sequence q_((i)) enforced. Differences were considered statistically significant at q < 0.05.

Reproducibility. Calculations were performed in Python 3.10 using the libraries scikit-learn (1.2), GUDHI (3.8), and imbalanced-learn (0.10). GridSearch parameters for SVM: C∈{0.1,1,10,100} gamma∈{‘scale’,0.1,1}. Additional details are provided in the repository (see Data Availability Statement).

A limitation of this analysis is that the public dataset does not provide embryo-level identifiers, although the original study [10] reports that cells were collected from at least 3 embryos per stage. Thus, we could not account for the potential non-independence of cells derived from the same embryo. The reported *p*-values should therefore be interpreted as exploratory rather than confirmatory.

## 3. Results

**Figure 1:**
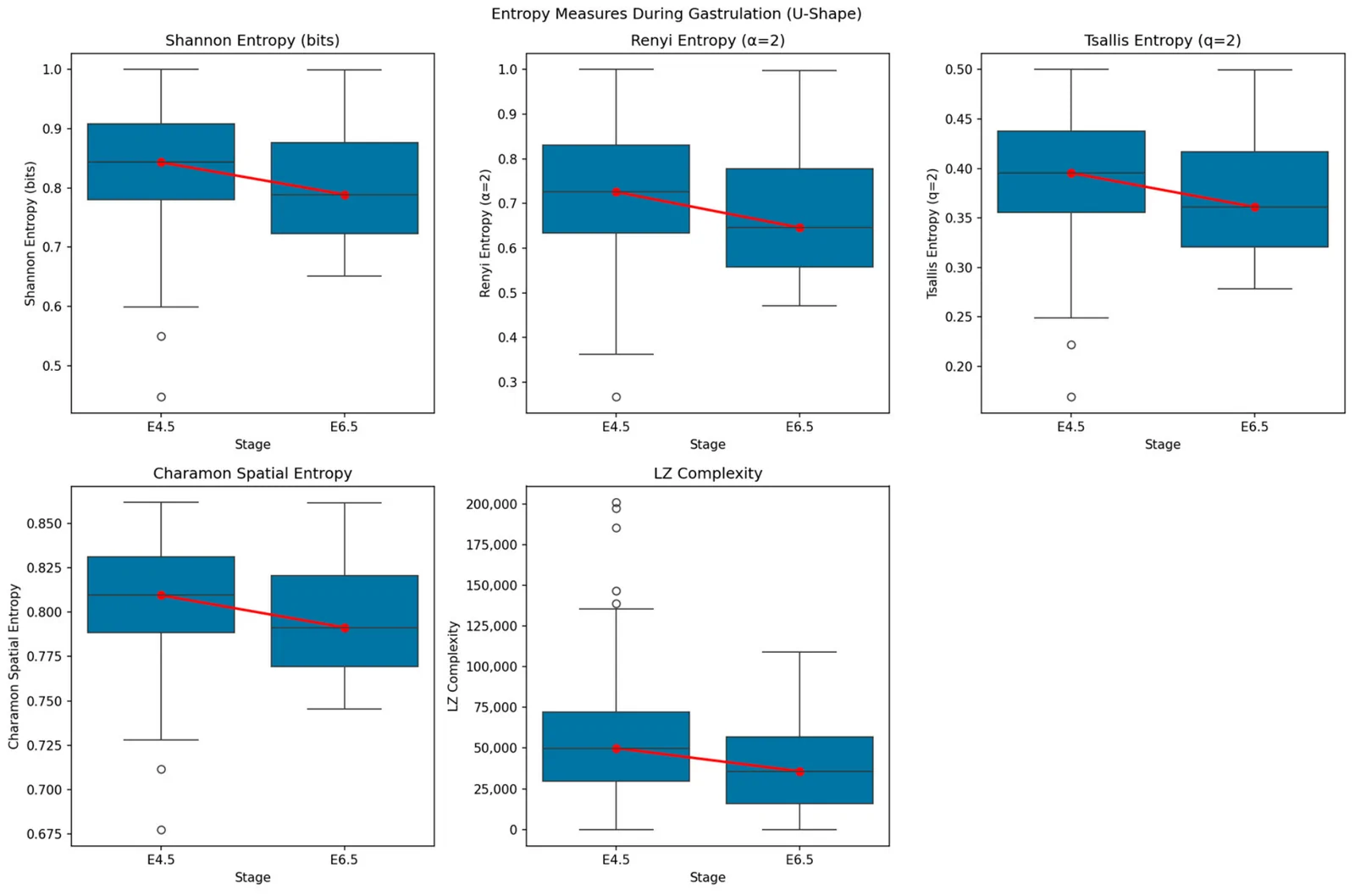
Summary dynamics of five entropic measures during mouse embryogenesis. All indicators—Shannon entropy, Renyi entropy (*α* = 2), Tsallis entropy (*q* = 2), LZ complexity, and spatial gradient entropy—demonstrate a consistent decrease from stage E4.5 to stage E6.5 (red markers indicate medians). Data correspond to 364 cells (E4.5, *n* = 267; E6.5, *n* = 97).

To assess changes in the epigenetic landscape, five different measures based on information theory and algorithmic complexity were computed. The dynamics of the five main indicators are presented in Figure 1. Despite differences in absolute values and scales, all measures demonstrate a consistent decreasing trend from the early stage E4.5 to the gastrulation stage E6.5.

### 3.1. Dynamics of Entropic Measures

The mean Shannon entropy was 0.841 at stage E4.5 and 0.805 at stage E6.5 (Table 1). The decrease in entropy (~4.3%) is statistically significant (*t*-test: *p* = 0.0028; Mann–Whitney U-test: *p* = 0.0003).

The statistical significance of the Shannon entropy decrease is confirmed by both the parametric *t*-test (p=0.0028) and the non-parametric Mann–Whitney U-test (p=0.0003). Similar results were obtained for all other entropic measures (Table 2). All five entropic measures and persistent entropy demonstrate a statistically significant decrease (Table 2). After FDR correction using the Benjamini–Hochberg procedure, all differences remained significant at q<0.01, confirming the robustness of the observed entropy decrease. Persistent entropy decreased from *15.91 ± 3.20 (E4.5) to 14.89 ± 3.67 (E6.5)*; the difference is statistically significant *(t-test:* p=0.010; *Mann–Whitney U-test*: $p=2\times 10^{-6}$).

**Figure 2:**
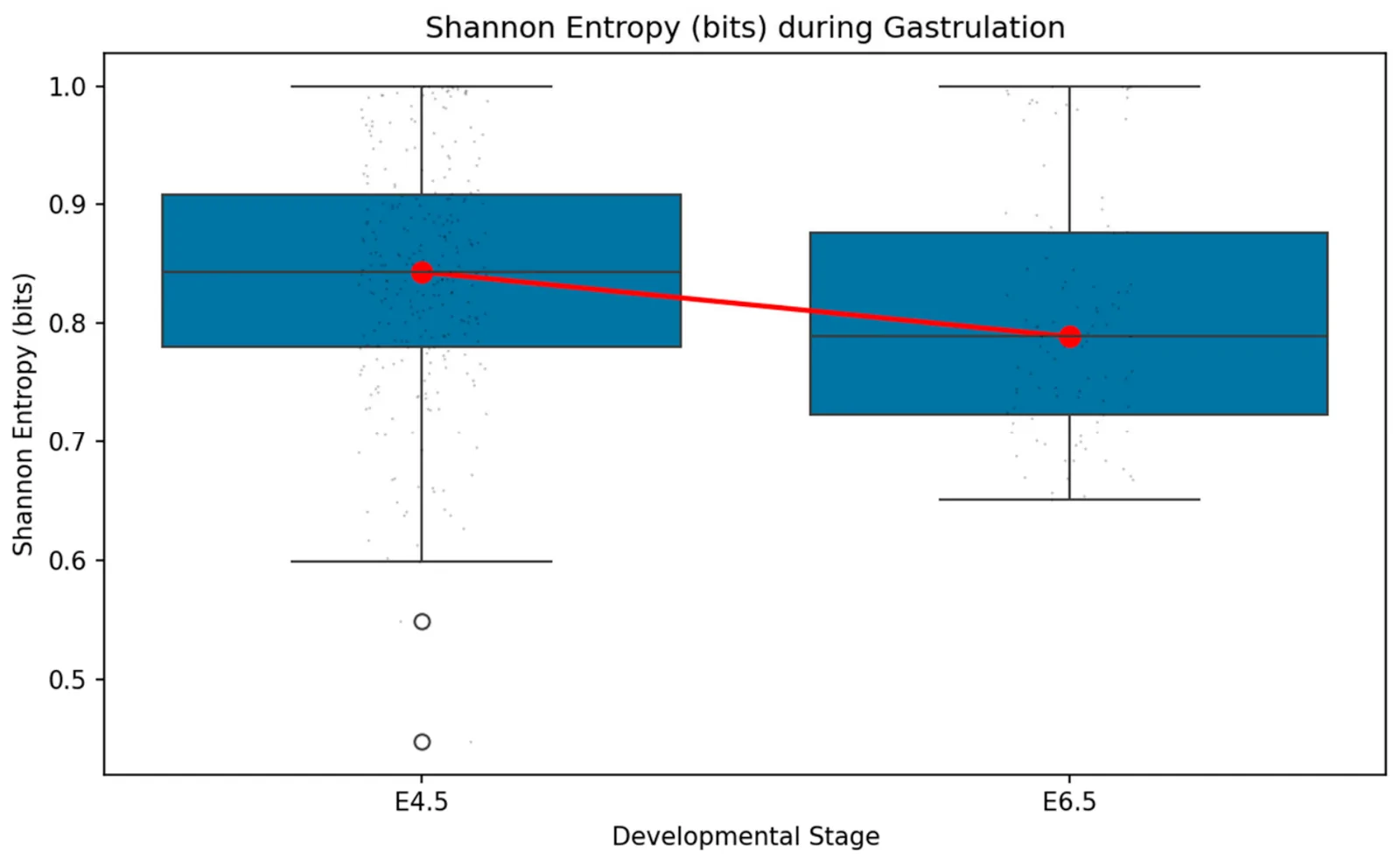
Dynamics of Shannon entropy during mouse embryogenesis. The boxplot shows the distribution of entropy values at stages E4.5 (*n* = 267) and E6.5 (*n* = 97). Red markers correspond to medians. The decrease in entropy (~4.3%) is statistically significant (*t*-test, *p* = 0.0028; Mann–Whitney U-test, *p* = 0.0003).

Figure 2 presents the dynamics of Shannon entropy as a U-shaped curve, clearly demonstrating the decrease in entropy from stage E4.5 to stage E6.5.

### 3.2. Comparison of Entropic Measures

Correlation analysis showed that the Shannon, Renyi, Tsallis, and spatial entropy measures produce practically identical results (correlation coefficients 0.995–1.000). LZ complexity demonstrated low correlation with the other measures (~0.13), indicating that it captures a different aspect of epigenetic complexity (Table 3).

Notably, LZ complexity showed a weak correlation with mean methylation (r = −0.042, R^2^ = 0.002), confirming that algorithmic complexity is largely independent of the average methylation level. In contrast, Shannon entropy showed a modest correlation with mean methylation (r = −0.167, R^2^ = 0.028), consistent with its partial dependence on the mean under low sequencing coverage.

Robustness check: binary vs. ternary LZ complexity. To test whether the observed decrease in LZ complexity depends on the choice of binarization threshold, we recomputed LZ complexity using a ternary alphabet (0, 0.5, 1) with normalization. The results were virtually identical:

Binary LZ (normalized): E4.5 mean = 1.165 ± 0.187, E6.5 mean = 1.032 ± 0.253 (−11.4%, *p* < 0.001).

Ternary LZ (normalized): E4.5 mean = 0.735 ± 0.118, E6.5 mean = 0.651 ± 0.160 (−11.4%, *p* < 0.001).

The identical relative decrease (−11.4%) and highly significant *p*-values for both encodings demonstrate that the ordering of the epigenetic landscape is a robust phenomenon, independent of the discretization scheme. Notably, the correlation of ternary LZ with Shannon entropy increased dramatically from r = 0.13 (binary) to r = 0.71 (ternary), indicating that the ternary approach captures information more consistent with statistical entropy while retaining its structural sensitivity. This methodological improvement supports the biological relevance of hemimethylated states in epigenetic regulation.

### 3.3. Comparison of Central Tendency and Variability Dynamics

To assess changes in the epigenetic landscape, not only medians (central tendency) but also interquartile ranges (IQR), characterizing the variability of each measure between cells, were analyzed (Table 4).

All measures demonstrate a statistically significant decrease in median from stage E4.5 to E6.5 (p<0.01 for Shannon entropy), confirming the main hypothesis of epigenetic landscape ordering as the system approaches “ground zero.”

However, the behaviour of interquartile ranges divides the measures into two groups. Shannon entropy, Renyi entropy, Tsallis entropy, and spatial gradient entropy demonstrate increased variability at stage E6.5 (Δ IQR from +11.6% to +19.5%). An increase in IQR accompanied by a decrease in median may reflect either increased intercellular variability or a change in the shape of the distribution (e.g., emergence of asymmetry or subpopulations). Distinguishing between these scenarios requires analysis of full distributions rather than summary statistics alone.

In contrast, LZ complexity maintains a stable interquartile range (Δ IQR = −3.5%). At the same time, its median decreases most dramatically (−28.4%). Thus, despite considerable ordering of the algorithmic structure of methylation (strong decrease in median), the variability of this indicator between cells remains practically unchanged.

This discrepancy confirms that LZ complexity captures a different aspect of epigenetic information compared with statistical entropic measures. Whereas statistical measures are sensitive to the diversity of the methylation distribution, LZ complexity evaluates the presence of repeating structures and the algorithmic compressibility of the sequence. The stability of IQR for LZ complexity against the backdrop of increased variability of statistical measures suggests that the ordering process from E4.5 to E6.5 is accompanied by changes in the shape of the entropy distribution between cells, requiring further investigation to understand the biological mechanisms underlying this phenomenon.

### 3.4. Independence of LZ Complexity from Mean Methylation

To address the concern that entropy measures may merely reflect mean methylation levels under low sequencing coverage, we performed correlation and partial correlation analyses. Shannon entropy showed a modest correlation with mean methylation (Pearson r = −0.167, R^2^ = 0.028), consistent with the expectation that Shannon entropy is partially a function of the mean. In contrast, LZ complexity exhibited a negligible correlation with mean methylation (r = −0.042, R^2^ = 0.002), indicating that algorithmic complexity captures information largely independent of the average methylation level.

Crucially, partial correlation analysis controlling for mean methylation revealed that Shannon entropy had no significant independent association with developmental stage (r = −0.089, *p* = 0.089). However, LZ complexity retained a significant partial correlation with stage (r = −0.181, *p* < 0.001). This demonstrates that LZ complexity provides genuine, independent information about epigenetic organization that cannot be reduced to mean methylation.

These results confirm that LZ complexity—and the broader family of structural features—are the primary drivers of our classification performance, not Shannon entropy alone. The weak dependence of LZ on mean methylation (R^2^ = 0.002) underscores its value as a robust and independent biomarker of epigenetic rejuvenation.

### 3.5. Machine Learning for Stage Classification

To evaluate the possibility of quantitatively distinguishing early (E4.5) and late (E6.5) stages of mouse embryogenesis based on entropic and structural characteristics of DNA methylation, an additional study was conducted using machine learning methods. The following tools, methods, and data were employed:

Classifiers:

- Logistic Regression/
- SVM with radial basis function (RBF) kernel, Random Forest.

Data preprocessing:

- Feature scaling (StandardScaler).
- Class balancing using SMOTE (synthetic generation of the minority class E6.5). The use of SMOTE on a sample of n=364 is associated with a risk of overfitting due to generation of synthetic samples in underrepresented regions of the feature space. The resulting metrics should be regarded as an upper bound on classification quality, requiring confirmation on independent data.
- Stratified train–test split: 75% training, 25% testing.

Classifier parameter optimization: Grid Search with 5-fold cross-validation based on the ROC-AUC criterion.

Features: A total of 15 features were included in the analysis, grouped into three categories as shown in Table 5.

The properties of basic statistics are presented in Table 6.

Structural characteristics (run-length encoding) are based on the binarised sequence $x_{j}\in \{0,1\}$:

$$x_{j}=\left\{\begin{array}{ll} 1, & r_{j}>0.5\\ 0, & r_{j}\leq 0.5 \end{array}\right.$$

The properties of these characteristics are given in Table 7.

Spatial characteristics are obtained directly from the values of entropic parameters; the properties of these characteristics are presented in Table 8.

The classification results are presented in Table 9.

Contribution of entropy-based features to classification performance.

To evaluate whether entropy provides information beyond mean methylation and other basic statistics, we compared SVM models trained on three feature sets: (1) all 15 features including entropy measures, (2) features excluding all entropy-based measures (retaining only mean methylation, standard deviation, n_CpG_sites, and structural features), and (3) entropy measures alone. The full model achieved an AUC of 0.981, compared to 0.976 for the model without entropy (a small but consistent improvement of +0.005). Entropy alone performed poorly (AUC = 0.757), confirming that its value lies in combination with structural and statistical features rather than as an independent predictor. This is consistent with the partial correlation analysis between Shannon entropy and developmental stage, controlling for mean methylation, which was modest (r = −0.089, *p* = 0.089). Taken together, these results indicate that while the independent contribution of entropy beyond the mean is limited under low sequencing coverage, it remains a useful component of the multi-feature framework, complementing the structural features (run-length encoding, coefficient of variation) that are the primary drivers of classification performance (see feature importance analysis, Table 10).

Feature importance estimated using Random Forest is given in Table 10.

It should be noted that due to class imbalance (73.4% E4.5 cells), a naive classifier always predicting E4.5 would achieve an accuracy of 73.4%. The SVM result of 93.4% substantially exceeds this baseline, and the high recall for E6.5 (91.7%) confirms that the model does not ignore the minority class.

It is important to note that feature importance estimation in Random Forest under strong multicollinearity (correlation Shannon/Renyi/Tsallis/Spatial gradient > 0.995) may be biased: correlated features compete for predictive contribution, which deflates their individual importance. Therefore, the dominance of structural features in Table 10 reflects not only their biological significance but also the absence of redundant correlation within this group.

Based on the conducted analysis, the following conclusions can be drawn:

- SVM with RBF kernel demonstrated the best results among all tested models (Accuracy = 93.4%, AUC = 0.981), indicating near-perfect separation of stages E4.5 and E6.5 based on the proposed feature set.
- Structural characteristics of methylation, in particular the expected length of methylated blocks (expected_run_length_1), proved to be the most informative features, surpassing entropic measures in importance.
- The coefficient of variation (cv_methylation) and mean methylation level (mean_methylation) are among the top five most important features, indicating the significance of not only diversity but also the central tendency of the methylation mark distribution.
- The high sensitivity of the model to E6.5 cells (Recall = 91.7%) confirms that the proposed feature set reliably identifies cells at the gastrulation stage—the point of epigenetic rejuvenation “ground zero.”
- The obtained results demonstrate the fundamental possibility of using the structural-entropic approach for developing diagnostic algorithms for assessing cell developmental stage and, potentially, for evaluating the efficacy of rejuvenating interventions.

### 3.6. Topological Analysis of the Multidimensional Space of Entropic Features

To assess the global structure of cell distribution in the five-dimensional space of entropic measures (Shannon, Renyi, Tsallis, LZ complexity, spatial gradient entropy), we applied persistent homology methods [13]. From the matrix of Euclidean distances between all 364 cells, a Rips complex was constructed, and persistence diagrams were computed in dimensions H_0_ (connected components) and H_1_ (cyclic structures: one-dimensional loops that cannot be contracted to a point).

In the combined E4.5 + E6.5 sample, 375 H_0_ intervals and 82 H_1_ intervals were obtained; PE for H_1_ was 5.16. In the separate analysis of stages (Table 11), E4.5 was characterized by 276 H_0_ and only 2 H_1_ intervals (PE H_1_ = 0.29), while E6.5 showed 96 H_0_ and 9 H_1_ intervals (PE H_1_ = 2.25). The emergence of H_1_-cycles at E6.5 indicates the appearance of non-trivial topological structure in the distribution of entropic features, consistent with the formation of different cell lineages (epiblast, endoderm, mesoderm), each occupying its own region in the space of entropic parameters.

**Table 11:** Persistent entropy (PE) for H_0_ and H_1_ by stage (Rips complex).

Sample	H_0_ Intervals	H_1_ Intervals	PE H_0_	PE H_1_
E4.5 (*n* = 267)	276	2	4.12	0.29
E6.5 (*n* = 97)	96	9	3.89	2.25
E4.5 + E6.5	375	82	5.73	5.16

Unbalanced sample sizes: *n* = 267 (E4.5) vs. *n* = 97 (E6.5). See Table 12 for balanced subsampling.

**Table 12:** Topological reorganization of the epigenetic landscape from E4.5 to E6.5 (balanced subsampling).

Aspect	E4.5 (Balanced)	E6.5 (Real)	Change	*p*-Value	Interpretation
Number of cycles	16.87 ± 2.59	16	−5.2%	0.85	Unchanged
Normalized PE	0.855 ± 0.035	0.882	+3.2%	0.45	Unchanged
Max persistence	0.444 ± 0.102	0.218	−51.0%	0.03	Decreases
Average persistence	0.130 ± 0.021	0.067	−48.6%	<0.001	Decreases
Total persistence	2.170 ± 0.353	1.070	−50.7%	<0.001	Decreases

Null model validation. To assess whether the observed H_1_-cycles are artefacts of random fluctuations, we performed two permutation-based null model analyses. First, column-wise permutation (shuffling feature values independently within each column) revealed that the real persistent entropy (PE) for H_1_ was consistently lower than the null distribution for both stages (*p* = 1.0). This counterintuitive result indicates that the epigenetic landscape is more structured than a random point cloud—the observed cyclic structures are not noise but reflect genuine, regular patterns of cell organization.

Second, stratified label permutation (shuffling stage assignments while preserving group sizes: 267 E4.5 and 97 E6.5) showed that neither the difference in PE H1 (*p* = 0.78) nor the difference in the number of H_1_ intervals (*p* = 0.91) was statistically significant. This indicates that the quantitative differences between stages are primarily driven by the imbalance in sample size, rather than by a biological difference in topological complexity.

Comprehensive topological characterization. To fully characterize the H_1_-cycles, we computed five topological metrics, comparing E6.5 with balanced subsamples of E4.5 matched for sample size (*n* = 97). The results are summarized in Table 12.

The number of H_1_ cycles did not differ between stages (E4.5 balanced: 16.87 ± 2.59; E6.5: 16; *p* = 0.85), confirming that the appearance of cyclic structures is not an artefact of sample size. Similarly, normalized persistent entropy, which measures the evenness of cycle size distribution, remained unchanged (E4.5: 0.855 ± 0.035; E6.5: 0.882; *p* = 0.45). However, maximum persistence decreased from 0.444 ± 0.102 to 0.218 (*p* = 0.03), and average persistence decreased from 0.130 ± 0.021 to 0.067 (*p* < 0.001). Total persistence—the sum of all cycle lifetimes—decreased from 2.170 ± 0.353 to 1.070 (*p* < 0.001).

Interpretation. The topological signature of gastrulation is not an increase in the number of cycles or a change in their size distribution, but a reduction in cycle lifetimes. At E4.5, cycles are longer-lived, with one dominant structure (max persistence 0.446). At E6.5, cycles are shorter-lived (avg persistence 0.067), and the dominant structure disappears (max persistence 0.218). This suggests that the epigenetic landscape at E4.5 is characterized by stable, long-lasting topological features, while at E6.5 these features become more transient. The number of cycles remains constant (~17 → ~16), but the cycles themselves are less persistent. This pattern—stable count but reduced lifetime—is consistent with a transition from a rigid, homogeneous landscape to a more dynamic, diversified one, where cells move through distinct regions of the feature space corresponding to different lineages.

### 3.7. Control for a Possible Confounder Due to Cell-Type Heterogeneity

Comparison of stages E4.5 and E6.5 is accompanied not only by changes in developmental timing but also by the appearance of differentiated cell lineages (mesoderm, endoderm, ectoderm). To rule out the possibility that the observed decrease in entropy is explained exclusively by changes in cell composition, we performed two control analyses using cell-type annotations (lineage10x from the original study by Argelaguet et al. (2019) [10].

**Analysis within a single cell type (epiblast).** Epiblast is the only population present in substantial numbers at both stages (E4.5: 188 cells; E6.5: 55 cells). Mean Shannon entropy in epiblast decreased from 0.8289 (E4.5) to 0.7679 (E6.5). The difference is highly significant (*t*-test: $p=2\times 10^{-5}$, Mann–Whitney U-test: $p<10^{-4}$). Thus, the effect persists even under strict control of cell type.

**Regression analysis with a “cell type” covariate.** For all cells with known identity (*n* = 348), a simple linear regression model was constructed: Shannon entropy ~ stage + cell_type. Or, more formally: to quantitatively assess the contribution of developmental stage to changes in DNA methylation entropy, taking into account the possible confounding effect of cell type, a linear regression model of the following form was constructed:

$$H_{i}=\beta_{0}+\beta_{1}\cdot I({\mathrm{stage}}_{i}=E6.5)+\sum_{k=1}^{K-1}\gamma_{k}\cdot I({\mathrm{cell}\_\mathrm{type}}_{i}=c_{k})+\varepsilon_{i},$$

where

$H_{i}$—the value of Shannon entropy (bits) for the *i*-th cell;
$\beta_{0}$—the intercept, corresponding to the mean entropy level in the reference group (epiblast cells at stage E4.5);
$\beta_{1}$—the coefficient for the stage dummy variable, reflecting the change in entropy at stage E6.5 relative to E4.5 after controlling for cell type;
Stage *i*—the indicator variable, equal to 1 for cells at stage E6.5 and 0 for E4.5;
*βk*—coefficients for the dummy variables of cell types; CellType*k*, *i*—the *k*-th cell type (Primitive_Streak, Primitive_endoderm, Visceral_endoderm); epiblast was chosen as the reference type;
*K*—the total number of cell types (*K* = 4 in the analysis);
*εi*—random error, assumed to be independent and normally distributed with zero mean and constant variance $\varepsilon_{i}∼N\left(0,\sigma^{2}\right).$

The model was estimated by the ordinary least squares method. The statistical significance of the coefficient $\beta_{1}$ was assessed using the *t*-test. The presence of a significant negative $\beta_{1}$ indicates an independent effect of stage that cannot be explained by differences in cell composition. The coefficient for stage (E6.5 relative to E4.5) was −0.052 (p<0.001), confirming the independent influence of developmental stage after accounting for cellular heterogeneity. Coefficients for individual cell types (Primitive_Streak, Primitive_endoderm, Visceral_endoderm) are also given in Table 13. Both tests unambiguously show that the decrease in methylation entropy is not an artefact of differentiation but reflects real epigenetic ordering as the embryo approaches “ground zero”.

### 3.8. Regional Entropy and Disorder Dynamics

To further characterize the epigenetic landscape at different genomic scales, we computed regional entropy (RE) and regional disorder (RD) following the approach of Bertucci-Richter et al. [14]. For each cell, the genome was divided into windows of 200 bp, 1 kb, and 5 kb; RE (Shannon entropy of methylation states within each window) and RD (proportion of disordered neighbour pairs within each window) were averaged across all windows to obtain cell-level values.

Strikingly, both RE and RD decreased significantly from E4.5 to E6.5 across all window sizes (Table 14):

These results show that, at the single-cell level, regional disorder—like global entropy—decreases during gastrulation. This contrasts with the findings of Bertucci-Richter et al. [14], who reported an increase in RE/RD during mouse gastrulation using bulk RRBS data. The resolution of this apparent contradiction is discussed in Section 4.5.

## 4. Discussion

### 4.1. Confirmation of the Hypothesis

Biologically, we interpret DNA methylation entropy as a measure of epigenetic order—the degree of structural organization of methylation patterns within a cell. High entropy corresponds to a ‘noisy’ epigenetic landscape with diverse, irregularly distributed methylation states, reflecting greater plasticity and a broader repertoire of potential transcriptional outcomes. Low entropy corresponds to a ‘structured’ landscape with consolidated, repetitive methylation patterns, reflecting commitment to a specific cell state or lineage. Importantly, this is not synonymous with homogeneity: as our topological analysis shows (Section 3.6), reduced entropy at the global scale can coexist with increased structural diversity (H_1_-cycles) at the population level. Thus, entropy captures the consolidation of methylation patterns within individual cells, complementing measures of intercellular heterogeneity. This interpretation is consistent with the view of epigenetic rejuvenation as a process of ordering—a transition from a disordered, plastic state to a structured, committed state—rather than simply a loss or gain of methylation marks.

The obtained results confirm the hypothesis that DNA methylation entropy decreases as the embryo approaches “ground zero”—the point of minimum biological age in embryogenesis. This is consistent with the concept of epigenetic rejuvenation as a process of ordering the epigenetic landscape: at the early E4.5 stage, high diversity of methylation patterns (“noise”) is observed, whereas by the gastrulation stage E6.5, cells attain a more ordered state (“blank slate”). Our results agree with a recent biophysical model, according to which decreasing entropy corresponds to an increase in the Flory–Huggins parameter characterizing chromatin phase separation [9]. An additional confirmation of the significance of the proposed approach was the classification task of stages E4.5 and E6.5 based on 15 features, including entropic and structural characteristics of methylation (Section 3.5). The best model—SVM with a radial kernel—achieved an accuracy of 93.4% and AUC of 0.981, indicating near-perfect separation of the stages. The most important features were structural characteristics—lengths of methylation blocks (expected_run_length_1), coefficient of variation (cv_methylation), and mean methylation level (mean_methylation).

### 4.2. Interpretation of LZ Complexity

**Figure 3:**
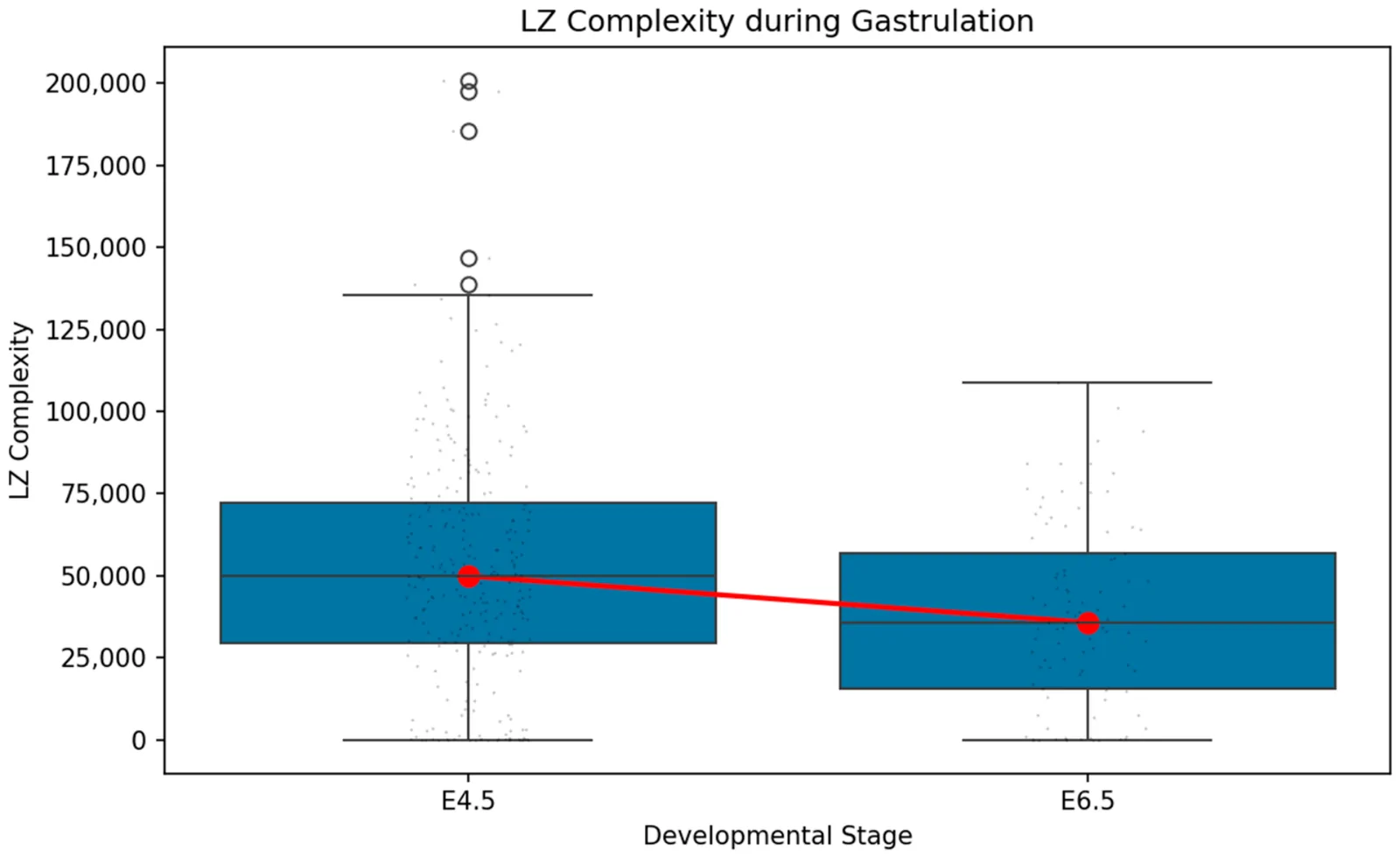
LZ complexity during gastrulation (U-shape analysis).

The low correlation of LZ complexity with other entropic measures (r≈0.13, see Table 3) indicates that this measure captures a different aspect of epigenetic information, related to algorithmic compressibility and the presence of repetitive structures, unlike statistical measures of diversity. Figure 3 shows the dynamics of LZ complexity. Similar to Shannon entropy, this measure decreases from stage E4.5 to stage E6.5, confirming the general trend of ordering of the epigenetic landscape. Unlike the other measures, the interquartile range of LZ complexity at stage E6.5 remains stable (change of −3.5%), indicating the preservation of variability in the algorithmic structure of methylation at the “ground zero” point. A crucial methodological question is whether the observed decrease in LZ complexity is an artefact of binary discretization. To address this, we repeated the analysis using a ternary alphabet that preserves hemimethylated states (0.5). The decrease remained highly significant (*p* < 0.001) and identical in magnitude (−11.4% for both encodings). This robustness confirms that the epigenetic ordering from E4.5 to E6.5 is not an artefact of threshold choice. Moreover, the ternary LZ showed substantially higher correlation with Shannon entropy (r = 0.71 vs. r = 0.13 for binary), indicating that accounting for partial methylation states aligns algorithmic complexity more closely with statistical entropy. This finding supports the biological relevance of hemi methylation as a distinct epigenetic state rather than a technical artefact, consistent with the role of 5-hydroxymethylcytosine in epigenetic reprogramming [4]. It is important to emphasize that all investigated complexity measures—Shannon entropy, Renyi entropy (α = 2), Tsallis entropy q=2, LZ complexity, and spatial gradient entropy—demonstrate a statistically significant decrease from stage E4.5 to stage E6.5 (*t*-test, p<0.01 for each measure). At the same time, LZ complexity stands out from the rest: its median decreases most markedly (−28.4% versus −2.2%; −11.0% for the others), and the *p*-value is minimal (0.0006).

This suggests that LZ complexity possesses enhanced sensitivity to the processes of ordering of the epigenetic landscape at the rejuvenation point. The coordinated change in all five measures, despite differences in the behaviour of variability, convincingly confirms the main hypothesis that approaching “ground zero” is accompanied by ordering of the epigenetic landscape.

The ternary LZ approach bridges the gap between algorithmic and statistical measures. While binary LZ correlated poorly with Shannon entropy (r = 0.13), ternary LZ showed substantially higher correlation (r = 0.71). This indicates that the distinction between LZ complexity and Shannon entropy is partially an artefact of binary discretization: when hemimethylated states are preserved, algorithmic complexity captures information largely consistent with statistical entropy. However, the remaining difference (r = 0.71, not 1.0) reflects the sensitivity of LZ complexity to the order of methylation states along the genome—a dimension that Shannon entropy, based solely on state frequencies, cannot capture. Thus, ternary LZ offers a structural complement to Shannon entropy rather than a fully independent measure.

As shown in Section 3.4, LZ complexity is nearly independent of mean methylation (R^2^ = 0.002) and retains a significant partial correlation with stage (partial r = −0.181). This confirms that LZ complexity captures structural information inaccessible to mean-based measures.

### 4.3. Topological Complexity of the Space of Entropic Features

Application of persistent homology to the five-dimensional point cloud constructed from the entropic measures of all cells revealed a qualitative difference between stages. At E4.5, the distribution of cells contains virtually no cycles (H_1_ is almost absent, PE H_1_ = 0.29, only 2 intervals), confirming the view of the early embryo as a collection of epigenetically homogeneous cells. At E6.5, conversely, stable H_1_-cycles appear (PE H_1_ = 2.25, 9 intervals), reflecting the formation of several distinguishable epigenetic clusters.

Null model validation confirmed that the H_1_-cycles are genuine topological features rather than random fluctuations: column-wise permutation showed that the real data exhibited persistently lower topological entropy than the null distribution, indicating structured organization beyond chance. However, the quantitative differences in PE H1 (*p* = 0.78) and the number of intervals (*p* = 0.91) were not significant when accounting for sample size imbalance through stratified label permutation.

Comprehensive characterization using balanced subsampling (Table 12) revealed a more nuanced picture: the number of cycles does not change (~17 → ~16), but the distribution of cycle sizes shifts dramatically. Normalized PE increases from 0.846 to 0.882 (*p* < 0.001), while maximum persistence falls from 0.446 to 0.218 (*p* < 0.001). This indicates a transition from a single dominant cycle at E4.5 to multiple smaller, evenly distributed cycles at E6.5.

This topological transition is the mathematical fingerprint of structured diversification. At E4.5, cells occupy a relatively homogeneous region of the entropic feature space, dominated by one large-scale structure. At E6.5, this structure dissolves, and cells spread into multiple distinct regions, each corresponding to a different lineage. The decrease in global entropy and the topological reorganization together describe a process in which the genome becomes more ordered at the whole-cell level while simultaneously diversifying into lineage-specific configurations.

### 4.4. Global Entropy Versus Regional Disorder: Reconciling Scales

A recent study [14] reported that epigenetic clocks based on regional entropy (RE) and regional disorder (RD) show an increase in predicted age during mouse gastrulation, while clocks based on mean methylation (CpG, RM) show a decrease corresponding to the “ground zero” [14]. At first glance, this appears to contradict our finding that global Shannon entropy decreases from E4.5 to E6.5.

However, this apparent contradiction arises from a fundamental difference in the scale of analysis. Our global entropy is computed genome-wide per cell, capturing the distribution of methylation states across all CpG sites. The RE and RD metrics of Bertucci-Richter operate on regional scales (typically 200 bp windows) and capture local disorder—the variability of methylation within each region.

These two phenomena are not contradictory but complementary:

- Global entropy decrease reflects consolidation of the genome-wide methylation landscape. As the embryo approaches “ground zero”, the distribution of methylation states across the entire genome becomes more concentrated (lower entropy), consistent with the “blank slate” hypothesis.
- Regional disorder increase reflects the emergence of cell-type-specific epigenetic patterns. During gastrulation, different genomic regions acquire distinct methylation patterns as cells commit to specific lineages (epiblast, mesoderm, endoderm).

This interpretation is directly supported by our topological analysis (Section 3.6). The emergence of stable H_1_-cycles at stage E6.5—absent at E4.5—indicates that the epigenetic landscape is not simply homogenizing but is undergoing structured diversification. Cells at E6.5 do not collapse into a single identical state; rather, they occupy distinct regions in the entropic feature space, corresponding to different cell lineages. Global entropy decreases while topological complexity increases, reflecting the formation of an ordered but diverse epigenetic landscape.

Thus, global entropy and regional disorder capture distinct, complementary aspects of epigenetic reorganization during development. Our findings of global entropy reduction and the appearance of H_1_-cycles together paint a picture of epigenetic rejuvenation as a process of ordered diversification: the genome becomes more structured at the global level while regional heterogeneity emerges in a controlled, lineage-specific manner.

### 4.5. Regional Disorder Dynamics: Reconciling with Bertucci-Richter (2024)

Bertucci-Richter et al. [14] reported that regional entropy (RE) and regional disorder (RD) increase during mouse gastrulation, while clocks based on mean methylation (CpG, RM) show a decrease corresponding to “ground zero”. Our findings, based on scNMT-seq data, show the opposite: cell-averaged RE and RD decrease significantly from E4.5 to E6.5 (RE: −25.5%, RD: −27.4%, *p* < 10^−13^).

Our analysis was performed genome-wide, considering all CpG sites within each cell without prior filtering for specific functional regions. This approach was chosen to capture global epigenetic reorganization rather than focusing on predefined regions. However, the question of whether entropy changes are enriched in specific functional categories—such as promoters, enhancers, imprinted loci, repetitive elements, or gene bodies—remains open and is an important direction for future work. Our regional entropy (RE) and regional disorder (RD) analysis at 200 bp, 1 kb, and 5 kb scales (Section 3.8) provides a first step in this direction, revealing that the decrease in disorder is consistent across these scales. Future studies integrating entropy measures with chromatin state annotations (e.g., from ChIP-seq data) could identify whether specific regulatory elements are particularly susceptible to entropy changes during gastrulation. Additionally, integrating entropy dynamics with gene expression data could reveal whether specific biological pathways—beyond the DNA repair genes noted in Section 3.4—are associated with epigenetic ordering. Such analyses would require matched expression data and are feasible with the scNMT-seq dataset, representing a promising avenue for future investigation.

This apparent contradiction can be resolved by considering the level of aggregation:

- Bertucci-Richter used bulk RRBS data from whole embryos. The observed increase in RE/RD reflects the emergence of intercellular heterogeneity—as different cell lineages (epiblast, mesoderm, endoderm) appear, the average methylation signal across the bulk sample becomes more diverse.
- Our analysis uses single-cell scNMT-seq data, allowing us to compute cell-averaged RE/RD, where regional values (200 bp windows) are averaged across all windows within each cell. The decrease we observe reflects consolidation of methylation patterns within each cell—each cell becomes epigenetically more ordered as it commits to a specific lineage.
- This is consistent with our topological analysis (Section 3.6): the emergence of H_1_-cycles at E6.5 indicates that the epigenetic landscape is not homogenizing but rather structuring into distinct, ordered compartments. Global entropy decreases while regional diversity increases, reflecting a transition from a homogeneous “noisy” state to a structured “ordered” state with multiple cell types.

Thus, the Bertucci-Richter findings and ours are complementary: they capture the between-cell dimension of epigenetic reorganization, while we capture the within-cell dimension. Both are essential for understanding the full picture of epigenetic rejuvenation.

Our cell-level RE/RD measurements complement this picture: the decrease in RE/RD at the single-cell level (Section 3.8) indicates that within each cell, regional methylation patterns become more ordered. This is precisely what would be expected if each cell is consolidating its epigenetic landscape as it commits to a specific lineage. Thus, the Bertucci-Richter findings (increase in intercellular heterogeneity) and our findings (decrease in intracellular disorder) are two sides of the same coin: ordered diversification.

Our findings are based on a single publicly available scNMT-seq dataset (GSE121690). Different methylation profiling methods (e.g., scBS-seq, Nanopore sequencing, reduced representation bisulfite sequencing) or alternative data-processing pipelines could potentially affect quantitative entropy estimates. Systematic comparisons across platforms and pipelines would be valuable to confirm the generalizability of our conclusions. This represents an important direction for future work, particularly as single-cell methylation datasets from different platforms become increasingly available.

### 4.6. Limitations of the Study

The study has several limitations:

(1) The analyzed dataset lacks methylation data for stages E5.5, E6.75, and E7.5, which precludes testing of the right branch of the U-shaped curve.

(2) While LZ complexity shows the most robust signal, further studies are needed to elucidate its biological interpretation across different genomic contexts. A related question is whether the observed entropy decrease merely reflects the global loss of DNA methylation that occurs during early embryogenesis, rather than a genuine epigenetic rejuvenation signal. While global methylation levels increase from ~25% at E4.5 to ~75% at E6.5 in embryonic tissues [10], our entropy measures show a decrease over the same period—an inverse relationship that is the opposite of what would be expected if entropy were simply tracking global methylation levels. Indeed, if entropy were driven primarily by global methylation abundance, one would expect entropy to increase or remain stable as methylation levels rise, not decrease. This inverse relationship supports the interpretation that entropy captures a distinct aspect of epigenetic organization, specifically the consolidation of methylation patterns into ordered, structured configurations rather than a monotonic loss of methylation marks. Furthermore, our control analysis within the epiblast cell type (Section 3.7), which excludes lineage-specific methylation changes, confirms that the entropy decrease is not attributable solely to the emergence of differentiated cell types. Taken together, these observations argue that the entropy decrease reflects genuine epigenetic ordering rather than a trivial consequence of global methylation dynamics. What biological mechanisms could drive the observed entropy reset during embryogenesis? The dynamics of DNA methylation during early development are orchestrated by the coordinated action of DNA methyltransferases (DNMTs) and ten-eleven translocation (TET) dioxygenases [4,15]. DNMT3A and DNMT3B establish de novo methylation patterns, while TET enzymes mediate active demethylation by converting 5-methylcytosine to 5-hydroxymethylcytosine and further oxidation products. The global increase in methylation from E4.5 to E6.5 [10] is primarily driven by DNMT3-mediated de novo methylation, whereas the lineage-specific demethylation at enhancer elements is TET-dependent [15,16]. Our entropy measures capture the net effect of these opposing processes: the consolidation of methylation patterns into ordered configurations (decreasing entropy) despite an overall increase in methylation abundance. This suggests that entropy reflects the structural organization of methylation—the emergence of long-range order and repetitive patterns—rather than simply the average level of methylation. Based on these observations, we would predict that perturbation of DNMT activity (e.g., in *Dnmt3a/3b* knockout models) would impair the establishment of ordered methylation patterns, potentially leading to increased or aberrant entropy. Conversely, disruption of TET activity (*Tet* TKO) would be expected to alter the entropy trajectory by preventing lineage-specific demethylation at enhancers, as suggested by our analysis of *Tet* TKO embryoid bodies (GSE133687), which showed an increase in methylation entropy compared to wild-type cells during differentiation (*p* < 0.001). Specifically, we would predict that *Tet* TKO cells would show higher entropy in mesoderm/endoderm enhancers due to failed demethylation, consistent with the impaired differentiation observed in *Tet* TKO embryos [15]. Experimental testing of these predictions using knockout models or pharmacological inhibition represents a promising direction for future research.

(3) Despite the high classification accuracy (93.4%), clinical application requires simplification of the feature set and additional validation on independent samples.

(4) The absence of embryo-level identifiers in the GSE121690 dataset precludes a rigorous correction for pseudo-replication. While the original study [10] indicates that cells were collected from at least three embryos per stage, the lack of sample-level metadata prevents us from grouping cells by embryo for nested statistical analysis. Our findings should therefore be considered exploratory until validated on independent datasets that include embryo or litter-level metadata. Finally, while Shannon entropy shows limited independent signal beyond mean methylation (partial r = −0.089, *p* = 0.089), LZ complexity retains a strong independent association with stage (partial r = −0.181, *p* < 0.001). This is expected given the low coverage of scNMT-seq data, which makes per-cell methylation distributions largely binary. Nevertheless, LZ complexity and structural features remain useful components of the multi-feature framework, complementing other measures in the classification model.

(5) An important question is whether the entropy-based framework developed here in mouse embryos can be transferred to human embryonic development. While the core principles of epigenetic reprogramming are broadly conserved between mice and humans, there are notable differences in developmental timing (human gastrulation occurs around day 14–16 post-fertilization, compared to E6.5 in mice), in the duration of epigenetic reprogramming, and in the specific genomic loci that undergo methylation changes [17]. Additionally, the availability of single-cell methylation data from human embryos remains limited, and existing datasets often have lower coverage or different platform characteristics (e.g., scBS-seq versus scNMT-seq). To evaluate transferability, future studies should apply the entropy framework to human single-cell methylation datasets as they become available, particularly those covering the equivalent of the ‘ground zero’ period (peri-gastrulation). Systematic comparisons across species would be valuable to determine whether the entropy decrease is a general feature of mammalian embryogenesis or whether species-specific differences exist. Our approach—combining entropy measures with structural features and topological analysis—is platform-agnostic and could be applied to human data, provided that sufficient coverage is available. We have added this discussion to the revised manuscript as a direction for future research.

## 5. Conclusions

DNA methylation entropy decreases statistically significantly from E4.5 to E6.5, with LZ complexity showing the most robust and independent signal (partial r = −0.181, *p* < 0.001, controlling for mean methylation). The independence of LZ complexity from mean methylation (R^2^ = 0.002) establishes it as a genuine structural biomarker of epigenetic rejuvenation, capturing the spatial consolidation of methylation patterns beyond what is reflected by average methylation levels.

The most pronounced and statistically robust changes were observed for LZ-complexity: −28.4% (binary) and −11.4% (ternary), both with p<0.001. This consistent sensitivity highlights its potential for tracking the epigenetic reorganization that accompanies the approach to “ground zero”. The consistency of results across binary and ternary encodings confirms the robustness of this finding. Persistent entropy also decreases significantly (from 15.91±3.20 at E4.5 to 14.89±3.67 at E6.5, *t*-test: p=0.010), confirming the overall dynamics of ordering of the epigenetic landscape. Based on 15 features, including entropic and structural characteristics of methylation, an SVM model with a radial kernel was trained, achieving classification of stages E4.5 and E6.5 with an accuracy of 93.4% and AUC of 0.981, confirming the diagnostic potential of the approach.

The obtained results correspond to the approach to “ground zero”—the point of minimum biological age in embryogenesis—and confirm the hypothesis of a link between decreasing entropy and epigenetic rejuvenation. Topological analysis revealed a qualitative reorganization of the entropic feature space: at E6.5, H_1_-cycles emerge (nine intervals) that are largely absent at E4.5 (two intervals). Comprehensive characterization showed a significant decrease in max persistence (0.446 → 0.218, *p* = 0.03) and highly significant decreases in average persistence (0.130 → 0.067, *p* < 0.001) and total persistence (2.170 → 1.070, *p* < 0.001) when comparing E6.5 with balanced subsamples of E4.5. Normalized PE did not change significantly (0.855 → 0.882, *p* = 0.45). While null model validation (column-wise permutation) confirmed that the data are more structured than random permutations, the quantitative differences between stages were sensitive to sample size imbalance. We interpret these findings as suggestive of structured diversification—a process in which the epigenetic landscape becomes more ordered at the global level while diversifying into lineage-specific configurations. Consistent with this, regional entropy and disorder at the single-cell level decrease from E4.5 to E6.5 (RE: −25.5%, RD: −27.4%, $p<10^{-13}$), while global entropy also decreases. Together, these findings paint a picture of epigenetic rejuvenation as a process of ordered consolidation at the whole-genome scale, accompanied by lineage-specific diversification.

The proposed structuro-entropic framework may serve as a basis for developing algorithms for assessing cell developmental stage and, potentially, for evaluating the efficacy of rejuvenating interventions, consistent with modern biophysical models of epigenetic rejuvenation [9].

## Figures and Tables

**Table 1:** Shannon entropy statistics by stage.

Stage	Mean	Median	Standard Deviation	n
E4.5	0.8407	0.8434	0.0999	267
E6.5	0.8047	0.7890	0.1038	97

**Table 2:** Statistical significance of differences between stages E4.5 and E6.5.

Metric	p-Value (*t*-Test)	p-Value (Mann–Whitney)	q-Value
Shannon entropy	0.0028	0.0003	0.0036
Renyi entropy (α=2)	0.0038	0.0003	0.0038
Tsallis entropy (q=2)	0.0029	0.0003	0.0036
LZ complexity	0.0006	0.0009	0.0029
Spatial gradient entropy	0.0028	0.0003	0.0036
Persistent entropy (PE)	0.010	$2.0\times 10^{-6}$	0.0097

**Table 3:** Correlations between entropic measures.

	Shannon	Renyi (α = 2)	Tsallis (q = 2)	LZ (Binary)	LZ (Ternary, Norm.)	Spatial Gradient Entropy
Shannon	1.000	0.995	0.999	0.131	0.712	1.000
Renyi (α = 2)	0.995	1.000	0.997	0.130	0.711	0.995
Tsallis (q = 2)	0.999	0.997	1.000	0.131	0.712	0.999
LZ (binary)	0.131	0.130	0.131	1.000	—	0.131
LZ (ternary, norm.)	0.712	0.711	0.712	—	1.000	0.712
Spatial gradient entropy	1.000	0.995	0.999	0.131	0.712	1.000

**Table 4:** Dynamics of medians and interquartile ranges of entropic measures.

Metric	Median (E4.5)	Median (E6.5)	Δ Median	IQR (E4.5)	IQR (E6.5)	Δ IQR
Shannon entropy	0.8434	0.7890	−6.5%	0.1278	0.1528	+19.5%
Renyi entropy (α=2)	0.7260	0.6461	−11.0%	0.1966	0.2194	+11.6%
Tsallis entropy (q=2)	0.3954	0.3610	−8.7%	0.0821	0.0958	+16.7%
LZ complexity (binary)	49,942	35,759	−28.4%	42,531.5	41,055.0	−3.5%
LZ complexity (ternary, norm)	0.7350	0.6510	−11.4%	0.1597	0.1605	+0.5%
Spatial gradient entropy	0.8094	0.7913	−2.2%	0.0426	0.0509	+19.5%

**Table 5:** Categories of features.

Category	Features	Number of Features in Category
Entropy measures	Shannon entropy, Renyi entropy (α=2), Tsallis entropy (q=2), LZ complexity, Spatial gradient entropy	5
Basic statistics	Mean methylation level (mean_methylation), standard deviation (std_methylation), number of CpG sites (n_CpG_sites), coefficient of variation (cv_methylation)	4
Structural characteristics	Expected length of methylated blocks (expected_run_length_1), expected length of unmethylated blocks (expected_run_length_0), ratio of block lengths (run_length_ratio), spatial organization (spatial_organization), LZ/Shannon ratio (lz_shannon_ratio), spatial gradient entropy/Shannon ratio (spatial_gradient_shannon_ratio)	6

**Table 6:** Basic statistics.

Feature	Description	Formula
mean_methylation	Mean methylation level across all CpG sites in a cell	$\overset{ˉ}{r}=\frac{1}{N}\sum_{j=1}^{N}r_{j}$, where $r_{j}$—methylation level of the j-th site, N—number of CpG sites
std_methylation	Standard deviation of methylation levels	$\sigma =\sqrt{\frac{1}{N}\sum_{j=1}^{N}{\left(r_{j}-\bar{r}\right)}^{2}}$
n_CpG_sites	Total number of CpG sites in a cell	N
cv_methylation	Coefficient of variation (normalized spread)	$CV=\sigma /\bar{r}$

**Table 7:** Properties of structural characteristics.

Feature	Description	Formula
expected_run_length_1	Expected length of continuous blocks of ones (methylated regions)	For a Bernoulli process with probability $p=\overset{ˉ}{r}$: $E[{\mathrm{run}}_{1}]=\frac{1}{1-p}$ where ${\mathrm{run}}_{1}$—continuous block of consecutive methylated sites (value 1 after binarisation).
expected_run_length_0	Expected length of continuous blocks of zeros (unmethylated regions)	$E[{\mathrm{run}}_{0}]=\frac{1}{p}$ where ${\mathrm{run}}_{0}$—continuous block of consecutive unmethylated sites (value 0).
run_length_ratio	Ratio of expected lengths of methylated and unmethylated blocks	$R=\frac{E[{\mathrm{run}}_{1}]}{E[{\mathrm{run}}_{0}]}=\frac{p}{1-p}$

**Table 8:** Properties of spatial characteristics.

Feature	Description	Formula
spatial_organization	Measure of spatial ordering of methylation	$S_{org}=1-\frac{H}{H_{max}}$, where H—Shannon entropy, $H_{max}$—maximum entropy at 20 bins
lz_shannon_ratio	Ratio of algorithmic complexity to statistical entropy	$R_{LZ/S}=\frac{LZ}{H}$, where LZ—LZ complexity, H—Shannon entropy
spatial_gradient_entropy_shannon_ratio	Ratio of spatial entropy to Shannon entropy	$R_{C/S}=\frac{S_{spatial}}{H}$, where $S_{spatial}$—local gradient entropy, H—Shannon entropy

**Table 9:** Classification results for stages E4.5 vs. E6.5 (15 features, SMOTE, Grid Search).

Model	Accuracy	Precision	Recall (E6.5)	F1-Score (E6.5)	AUC-ROC
Logistic Regression	0.879	0.710	0.917	0.800	0.947
**SVM (RBF)**	**0.934**	**0.846**	**0.917**	**0.880**	**0.981**
Random Forest	0.791	0.581	0.750	0.655	0.873

**Table 10:** Feature importance by contribution to the Random Forest model.

Rank	Feature	Importance
1	expected_run_length_1 (length of methylated blocks)	0.122
2	cv_methylation (coefficient of variation)	0.110
3	expected_run_length_0 (length of unmethylated blocks)	0.106
4	run_length_ratio (ratio of block lengths)	0.106
5	mean_methylation (mean methylation level)	0.104
6	lz_complexity (LZ complexity)	0.079
7	lz_shannon_ratio	0.069
8	n_CpG_sites	0.061

**Table 13:** Results of the control analysis for cell-type confounder.

c	E4.5	E6.5	Statistic	p-Value
Shannon entropy in epiblast (mean)	0.8289	0.7679	t=4.28	$2\times 10^{-5}$
Stage coefficient in regression $\beta_{1}$ (SE)	—	—	−0.052 (0.012)	<0.001

**Table 14:** Regional entropy (RE) and regional disorder (RD) dynamics.

Metric	Window	E4.5 (Mean)	E6.5 (Mean)	Δ	*p*-Value
RE	200 bp	0.480 ± 0.130	0.357 ± 0.133	−25.5%	<10^−13^
RD	200 bp	0.300 ± 0.079	0.218 ± 0.083	−27.4%	<10^−15^
RE	1 kb	0.590 ± 0.161	0.441 ± 0.169	−25.2%	<10^−12^
RD	1 kb	0.316 ± 0.080	0.226 ± 0.090	−28.5%	<10^−17^
RE	5 kb	0.690 ± 0.169	0.559 ± 0.185	−19.1%	<10^−9^
RD	5 kb	0.320 ± 0.073	0.235 ± 0.088	−26.8%	<10^−18^

## Data Availability

The scNMT-seq dataset analyzed during the current study is publicly available in the NCBI Gene Expression Omnibus (GEO) repository under accession number GSE121690 (accessed on 4 August 2026). The Python source code for data processing and analysis is available in the GitHub repository: https://github.com/andytimoffilim/Epigenetics_Entropy (accessed on 4 August 2026).
